# Sustaining HIV service delivery to key population clients using client‐centered models during the debate and enactment of the Anti‐Homosexuality Act in Uganda

**DOI:** 10.1002/jia2.26253

**Published:** 2024-05-07

**Authors:** Vamsi Vasireddy, Natalie E. Brown, Neha Shah, Trevor A. Crowell

**Affiliations:** ^1^ U.S. Military HIV Research Program, Walter Reed Army Institute of Research Silver Spring Maryland USA; ^2^ U.S. Mission to Uganda Kampala Uganda; ^3^ U.S. Department of State and Smithsonian Institution Washington DC USA; ^4^ Henry M. Jackson Foundation for the Advancement of Military Medicine, Inc. Bethesda Maryland USA

1

Punitive and discriminatory laws against key populations (KP), particularly men who have sex with men (MSM), have been on the rise for over a decade [1, 2]. Studies have shown these laws to be associated with healthcare avoidance, decreased HIV testing and increased HIV prevalence [3, 4, 5]. These laws further marginalize groups that are disproportionately affected by HIV [6‐7] and imperil the achievement of the UNAIDS 95‐95‐95 targets [8].

In Uganda, same‐sex relationships have been illegal since the early 20th century. In 2010, a newspaper infamously outed 100 alleged homosexuals, driving many into hiding [9]. The 2014 Anti‐Homosexuality Act (AHA), colloquially known as the “Kill the Gays” bill, penalized consensual same‐sex sexual acts with death or life imprisonment, but was struck down by the Constitutional Court. Ugandan media started discussing a possible new AHA in December 2022. On 21 March 2023, Parliament overwhelmingly passed a new AHA, which was signed into law on 26 May 2023. The 2023 AHA criminalizes same‐sex sexual acts with sentences ranging from 10‐year imprisonment to death. It also criminalizes the promotion of homosexuality, which is broad enough to include routine public health activities, such as HIV prevention, safer sex education and community engagement. This creates opportunities for abuse, puts organizations delivering healthcare to MSM in danger of prosecution and impedes access to KP‐friendly services. On 3 April 2024, following a lengthy legal challenge, the Ugandan Constitutional Court upheld the majority of the AHA, leaving in place problematic sections of AHA including the prohibition against “promotion of homosexuality,” the use of the death penalty for repeat offenders engaging in consensual sexual contact and allowing for “rehabilitation” of LGBTQI+ persons.

The United States President's Emergency Plan for AIDS Relief (PEPFAR) supports over 1.3 million Ugandans on antiretroviral therapy (ART). In 2023, PEPFAR supported 84 drop‐in‐centres (DICs) across Uganda that provided comprehensive HIV prevention and treatment services for KP clients. The DICs were unnamed/unidentified to create confidential and safe spaces for KP clients, including MSM, female sex workers (FSWs) and transgender persons. DICs were strategically situated for easy access and staffed by at least one nurse and a mix of community health workers, including peers within the KP communities. Service delivery data from DICs were de‐identified, disaggregated by type of KP clients and services, and uploaded to a central database. Following concerns raised by KP clients regarding healthcare access, we initiated monitoring of AHA impacts on HIV service delivery and implemented new adaptations to support care delivery.

This report focuses on three DICs operated by a single agency that provided consistent data and served a representative population of KP clients. A larger sample was not possible due to inconsistent data access and the sensitive nature of DIC locations and operations. We noticed a steep decline in DIC visits coinciding with escalating anti‐homosexual sentiment and reporting in the media (Figure 1). At least four DICs closed during this time due to safety incidents. Numerous MSM reported evictions from their residences and assault. The PEPFAR programme quickly pivoted its models to support KP clients as described below.

**Figure 1:**
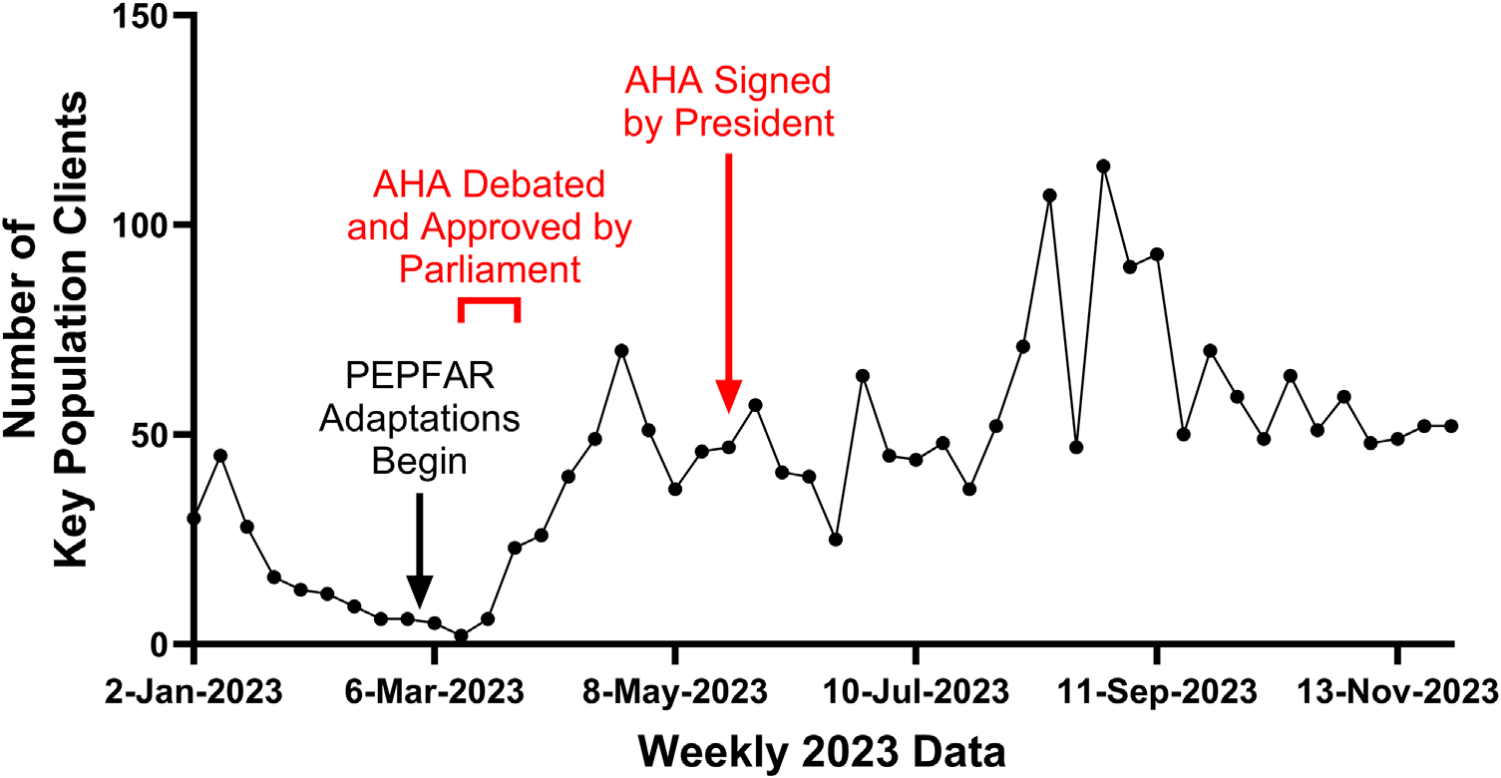
Weekly cumulative service delivery visits at three Ugandan drop‐in‐centres during debate and enactment of the Anti‐Homosexuality Act. Number of clients served at three Ugandan drop‐in centres are reported on a weekly basis, starting on Mondays. Between January and April 2023, a dramatic decline in visits was observed, which reversed as service delivery adaptations were implemented. Weekly visits subsequently continued at a steady pace, similar to baseline, through November 2023.

Innovative approaches:

- **Person‐centred telehealth interventions**. Telehealth models introduced during the COVID‐19 pandemic were adapted by peer counsellors who called clients to remind them of their ART or Pre‐exposure prophylaxis (PrEP) refills, conduct adherence counselling and offer information about resources available to evicted or displaced clients. Telehealth was not counted as a DIC visit, but most telehealth clients eventually returned to the DICs.
- **Reinforced safety measures**. DICs that had experienced break‐ins and violence hired security guards, installed lockable cabinets for client files and installed security cameras.
- **Enhanced data security**. All PEPFAR‐supported implementing partners (IPs) introduced enhanced data security measures such as locked offices and fingerprint access to data storage rooms. PEPFAR Uganda developed a data security policy to respond to potential data breaches.

Adapting and scaling differentiated service delivery models:

- **Home delivery**. Home delivery of ART and prevention products, like condoms and PrEP, was conducted by peer counsellors who are trusted community members. Community workers were sensitized on safety and protecting client confidentiality during deliveries.
- **Multi‐month dispensing**. The pre‐existing initiative to dispense 3‐ or 6‐months of ART to eligible clients was rapidly scaled up.
- **Community‐based viral load monitoring**. For clients afraid to visit health facilities for routine viral load testing, a model was established wherein a nurse would go into the community and draw blood from clients. While this model was useful for some, it was expensive and not widely utilized because of clients’ reluctance to disclose their places of residence or work due to safety concerns.
- **Community drug dispensing points (CDDPs)**. Peer counsellors and community health workers carried ART and PrEP into the community and dispensed to eligible clients. CDDPs were in safe, discreet and trusted areas of the community. Some CDDPs were located within brothels to facilitate access by FSWs, though other KP clients could access services as well.
- **Paralegal support**. Paralegals were employed to support KP clients and strengthen community trust by providing information on the rights and protections under Ugandan laws. Paralegals directed victims to resources for further legal assistance.

Process of adapting the programmes:

- The PEPFAR programme designed and implemented the adaptations following extensive discussions–and in collaboration–with Ugandan civil society organizations (CSOs), IPs and the US government.
- Numerous consultations were held with the community and civil society, which are ongoing, because the programme adaptations are dynamic and responsive to the ever‐changing political context in Uganda.
- Weekly and ad hoc consultations were held with the KP clients, CSOs and IPs. Communities and CSOs were concerned about accessing KP‐friendly services due to safety concerns. In response, PEPFAR scaled‐up physical security features, instituted data protection measures, stopped collecting/reporting identifiable data and continues to review the programme with the beneficiaries.
- PEPFAR implementing agencies are in constant communication with each other and the bureau of Global Health Security and Diplomacy, to review and advocate for additional help.
- No additional funding was needed for these programme adaptations as PEPFAR implementing agencies found efficiencies within existing programmes and re‐purposed funds to strengthen KP‐friendly services.

With these programme adaptations, the DICs analysed here started seeing a return of KP clients that was maintained through the end of observation in November 2023. However, anecdotally, the effect has not been consistent across all DICs and others have not seen the same return of clients. The DICs are located across Uganda with varying community engagement and resistance. Some communities did not want data reported from the DICs due to fear of identification. PEPFAR respected these community preferences. Support from local governments also varied across the country, with some district leadership and police actively opposing DIC operations.

As KP communities continue to face stigma, discrimination and punitive legislation, it is important that PEPFAR adapt to maintain the delivery of life‐saving healthcare services. PEPFAR continues to monitor service delivery and utilization weekly to ensure quick adaptation of services, despite the negative impacts of the AHA. Our experience in Uganda can inform efforts to maintain resilient healthcare systems and services for KP clients in the face of growing punitive and discriminatory legislations in other countries. Countries facing such situations might employ similar monitoring procedures and implement similar programme adaptations to mitigate the impact of harmful legislations. Community engagement and inputs are critical for KP‐friendly services that reach the clients through outreach methods if the clients are fearful of accessing a health facility. Programmes and funders must be flexible to quickly adapt service delivery initiatives without red tape. Lastly, high‐level advocacy from funders and diplomatic partners should continue for upholding human rights and equitable healthcare access for all.

## COMPETING INTERESTS

The authors have no competing interests to declare.

## AUTHORS’ CONTRIBUTIONS

VV conceived of this work, conducted the analyses and authored the original draft of the manuscript. NEB provided resources and methodologic input. NS provided administrative support to the project, methodologic input and supervision of the work. TAC provided methodologic input and supervision of the work. All authors assisted in the writing, review, and editing of the manuscript and approved the manuscript for publication in its final form.

## FUNDING

This work was supported by the President's Emergency Plan for AIDS Relief via a cooperative agreement between the Henry M. Jackson Foundation for the Advancement of Military Medicine, Inc. and the US Department of Defense (W81XWH‐18‐2‐0040).

## DISCLAIMER

The views expressed are those of the authors and should not be construed to represent the positions of the US Army, the Department of Defense, the Department of State or the Henry M. Jackson Foundation for the Advancement of Military Medicine.

## PRIOR PRESENTATION

This work was presented, in part, at the 12th IAS Conference on HIV Science in Brisbane, Australia, 23–26 July 2023.

## Data Availability

The data that support the findings of this study are available on request from the corresponding author. The data are not publicly available due to privacy or ethical restrictions.

## References

[1] Davis SL , Goedel WC , Emerson J , Guven BS . Punitive laws, key population size estimates, and Global AIDS Response Progress Reports: an ecological study of 154 countries. J Int AIDS Soc. 2017;20(1):21386. 28364567 10.7448/IAS.20.1.21386 PMC5467607

[2] Editorial Board . It's not just Uganda. Much of Africa is marching backward on LGBT rights. Washington, DC: Washington Post. [Updated 2023 April 2; cited 2023 December 17]. Available from: https://www.washingtonpost.com/opinions/2023/04/02/uganda‐africa‐lgbt‐intolerance‐bill/

[3] Stannah J , Dale E , Elmes J , Staunton R , Beyrer C , Mitchell KM , et al. HIV testing and engagement with the HIV treatment cascade among men who have sex with men in Africa: a systematic review and meta‐analysis. Lancet HIV. 2019;6(11):e769–e787. 31601542 10.1016/S2352-3018(19)30239-5 PMC6993044

[4] Lyons CE , Twahirwa Rwema JO , Makofane K , Diouf D , Mfochive Njindam I , Ba I , et al. Associations between punitive policies and legal barriers to consensual same‐sex sexual acts and HIV among gay men and other men who have sex with men in sub‐Saharan Africa: a multicountry, respondent‐driven sampling survey. Lancet HIV. 2023;10(3):e186–e194. 36623537 10.1016/S2352-3018(22)00336-8 PMC10288909

[5] Schwartz SR , Nowak RG , Orazulike I , Keshinro B , Ake J , Kennedy S , et al. The immediate effect of the Same‐Sex Marriage Prohibition Act on stigma, discrimination, and engagement on HIV prevention and treatment services in men who have sex with men in Nigeria: analysis of prospective data from the TRUST cohort. Lancet HIV. 2015;2(7):e299–e306. 26125047 10.1016/S2352-3018(15)00078-8 PMC4481876

[6] Hessou PHS , Glele‐Ahanhanzo Y , Adekpedjou R , Ahouada C , Johnson RC , Boko M , et al. Comparison of the prevalence rates of HIV infection between men who have sex with men (MSM) and men in the general population in sub‐Saharan Africa: a systematic review and meta‐analysis. BMC Public Health. 2019;19(1):1634. 31801503 10.1186/s12889-019-8000-x PMC6894288

[7] Sandfort TGM , Dominguez K , Kayange N , Ogendo A , Panchia R , Chen YQ , et al. HIV testing and the HIV care continuum among sub‐Saharan African men who have sex with men and transgender women screened for participation in HPTN 075. PLoS One. 2019;14(5):e0217501. 31150447 10.1371/journal.pone.0217501 PMC6544251

[8] The Path That Ends AIDS: 2023 UNAIDS Global AIDS Update. Geneva: Joint United Nations Programme on HIV/AIDS; 2023.

[9] Rice X . Ugandan paper calls for gay people to be hanged. London: The Guardian. [Updated 2010 October 21; cited 2023 December 17]. Available from: https://www.theguardian.com/world/2010/oct/21/ugandan‐paper‐gay‐people‐hanged

